# Phosphorylated α-synuclein deposited in Schwann cells interacting with TLR2 mediates cell damage and induces Parkinson’s disease autonomic dysfunction

**DOI:** 10.1038/s41420-024-01824-8

**Published:** 2024-01-26

**Authors:** Yangxia Li, Qing Tong, Ye Wang, Yue Cheng, Yao Geng, Tian Tian, Yongsheng Yuan, Yi Fan, Ming Lu, Kezhong Zhang

**Affiliations:** 1 Department of Neurology, The First Affiliated Hospital of Nanjing Medical University, Nanjing, China; 2 Jiangsu Key Laboratory of Neurodegeneration, Department of Pharmacology, Nanjing Medical University, Nanjing, China

**Keywords:** Parkinson's disease, Movement disorders

## Abstract

Despite the significant frequency of autonomic dysfunction (AutD) in Parkinson’s disease (PD) patients, its pathogenesis and diagnosis are challenging. Here, we aimed to further explore the mechanism of phosphorylated α-synuclein (p-α-syn) deposited in vagus nerve Schwann cells (SCs) causing SCs damage and PD AutD. 1-methyl-4-phenyl-1,2,3,6-tetrahydropyridine (MPTP, 20 mg/kg) was administrated to C57BL/6 mice twice a week for 35 days. Following the final injection, locomotor functions, gastrointestinal symptoms, urine functions, and cardiovascular system functions were evaluated. Meanwhile, we examined p-α-syn deposited in vagus nerve SCs, Toll-like receptor 2 (TLR2) activation, and SCs loss using immunofluorescence, western blot, and Luxol fast blue staining. In vitro, the rat SCs line RSC96 cells were exposed to α-synuclein preformed fibril (α-syn PFF), and cell viability was detected by CCK8. Co-IP was used to identify the interaction between p-α-syn and TLR2. Furthermore, the role of TLR2 in p-α-syn-mediated SCs damage was confirmed by the administration of CU-CPT22, a specific blocker of TLR2. In vivo, apart from dyskinesia, MPTP mice exhibited constipation, urinary dysfunction, and cardiovascular failure, which were associated with the deposition of p-α-syn in vagus nerve SCs, TLR2 activation, and vagus nerve demyelination. In vitro, stimulation of α-syn PFF induced a time-dependent loss of viability, and p-α-syn deposited in RSC96 cells induced a cellular inflammatory response by interacting with TLR2, resulting in cell dysfunction and apoptosis. However, both SCs inflammatory response and cell viability were alleviated after inhibition of TLR2. Furthermore, 1 h fecal pellets and water content, the frequency of 1 h urine, blood pressure, heart rate, and heart rate variability of mice in the MPTP + CU-CPT22 group were also improved. Our results support the perspective that p-α-syn interacts with TLR2 induced SCs damage and is involved in PD AutD, which sheds fresh light on the mechanism of PD AutD and indicates a promising treatment for PD AutD targeting SCs p-α-syn/ TLR2 signaling pathway.

## Introduction

Advances in understanding the extent and effect of Autonomic dysfunction (AutD) in Parkinson’s disease (PD) have increased significantly over the past decade [1]. Patients with PD may exhibit AutD in the prodromal phase consisting of gut dysfunction, underactive bladder, orthostatic hypotension, and cardiovascular dysfunction, which gets more common as the disease progresses, severely affecting the quality of life and increasing mortality [2]. Despite the high prevalence of AutD in PD, clinical treatment is not significant, and lack of effective means of prevention [3]. Of note, the pathology and mechanism of AutD in PD are still poorly understood. Coon et al. proposed that AutD in synucleinopathies is the result of different involvement of central and peripheral autonomic networks [4]. Therefore, further clarification of AutD pathophysiology is critical for targeted therapy and prevention of PD progression.

The typical pathological hallmarks of PD include Lewy bodies and Lewy neurites that are mostly composed of phosphorylated α-synuclein (p-α-syn) [5]. Recent studies have noted that p-α-syn is not merely accumulated in the central nervous system (CNS), but also involved in multiple peripheral nervous systems (PNS) including the intestine, submandibular gland, and skin [6–8]. In our previous study, the deposition of p-α-syn in the sural nerves of PD patients was up to 100% and was mainly detected in Schwann cells (SCs) [9]. Moreover, p-α-syn was also detected in the sciatic nerve of 1-methyl-4-phenyl-1,2,3,6-tetrahydropyridine (MPTP)-induced PD mice which induced electrophysiological damage to sciatic nerves indicating the critical role of aberrant deposition of p-α-syn in peripheral nerve dysfunction [10].

According to Braak’s hypothesis, there is growing evidence demonstrating that the vagus nerve is a conduit for the spread of PD pathology from the periphery to the dorsal motor nucleus of the vagus in the brainstem [11]. Vagotomy partially halts the transfer of p-α-syn from the enteric nervous system to the substantia nigra and improves nonmotor symptoms, suggesting autonomic nerves may be the source of PD pathology and a possible target [6]. Furthermore, the latest study has demonstrated that stimulation of the afferent vagus nerve improves behavioral deficits and reduces the infiltration of inflammatory glial cells in the substantia nigra in rats with PD [12]. Indeed, our previous studies confirmed that the vagus nerve of rats overexpressing human mutated A53T α-synuclein induced prominent signs of AutD, accompanied by disrupted myelin sheaths [13]. All of these findings point out that the vagus nerve is a potential target for peripheral therapies which could prevent the transmission of α-synuclein from the periphery to the central and improve motor and non-motor deficits.

SCs are peripheral glial cells that constitute the myelin sheathes of the vagus nerve, acting as the oligodendrocyte homologous cells in the CNS. The insulating property of myelin sheathes might contribute to the reduction of membrane capacitance and enhancement of nerve conduction velocity [14]. Once this well-organized system is damaged, the functions of the PNS could be influenced and then peripheral neuropathy may occur. Our prior publications illuminated the damage to SCs in the sural nerves of PD patients, the sciatic nerve of MPTP mice, and the vagus nerve of rats overexpressing human mutated A53T α-synuclein, manifested as SCs swelling and numerous lipid vacuoles, additionally, rats injected with AAV-A53T showed reduced intestinal blood flow and electrophysiological damage accompanied by constipation symptoms preceding the occurrence of movement disorders [9, 10, 13]. Although all of the preceding investigations found evidence of SCs damage in PD peripheral nerves, the underlying mechanisms and their possible role in PD AutD remain to be determined.

As a family of pattern recognition receptors, Toll-like receptors (TLRs) initiate immune responses by identifying cell damage-associated molecular patterns (DAMPs) [15], while being able to regulate endogenous misfolded proteins released following exogenous pathogens and cellular stress. TLR2 is broadly expressed in neurons, astrocytes, microglia, and SCs. A study has shown that anti-TLR2 administration alleviated α-synuclein accumulation, neuroinflammation, and neurodegeneration in neurons and astrocytes in the α-synuclein high expression mouse model of PD [16]. The α-synuclein released by neurons acts as an endogenous agonist of TLR2, activating the inflammatory response in microglial [17]. Previously, sequencing of the sciatic nerve of chronic PD mice treated with MPTP showed that TLR2 was significantly upregulated in the MPTP group compared to the saline group [10]. Furthermore, the knockdown of TLR2 alleviated vagus nerve demyelination and improved gastrointestinal motility disorder in AAV-A53T rats [13]. Nevertheless, the involvement of TLR2 in vagus nerve SCs damage and consequent AutD in PD needs to be further investigated.

Therefore, we hypothesized that p-α-syn deposited in vagus nerve SCs and mediated SCs damage by interacting with TLR2, then inducing PD AutD. Overall, our study provides and justifies new targets for the treatment of PD AutD.

## Results

### AutD and central dopaminergic (DA) neurodegeneration in MPTP-treated mice

**Fig. 1:**
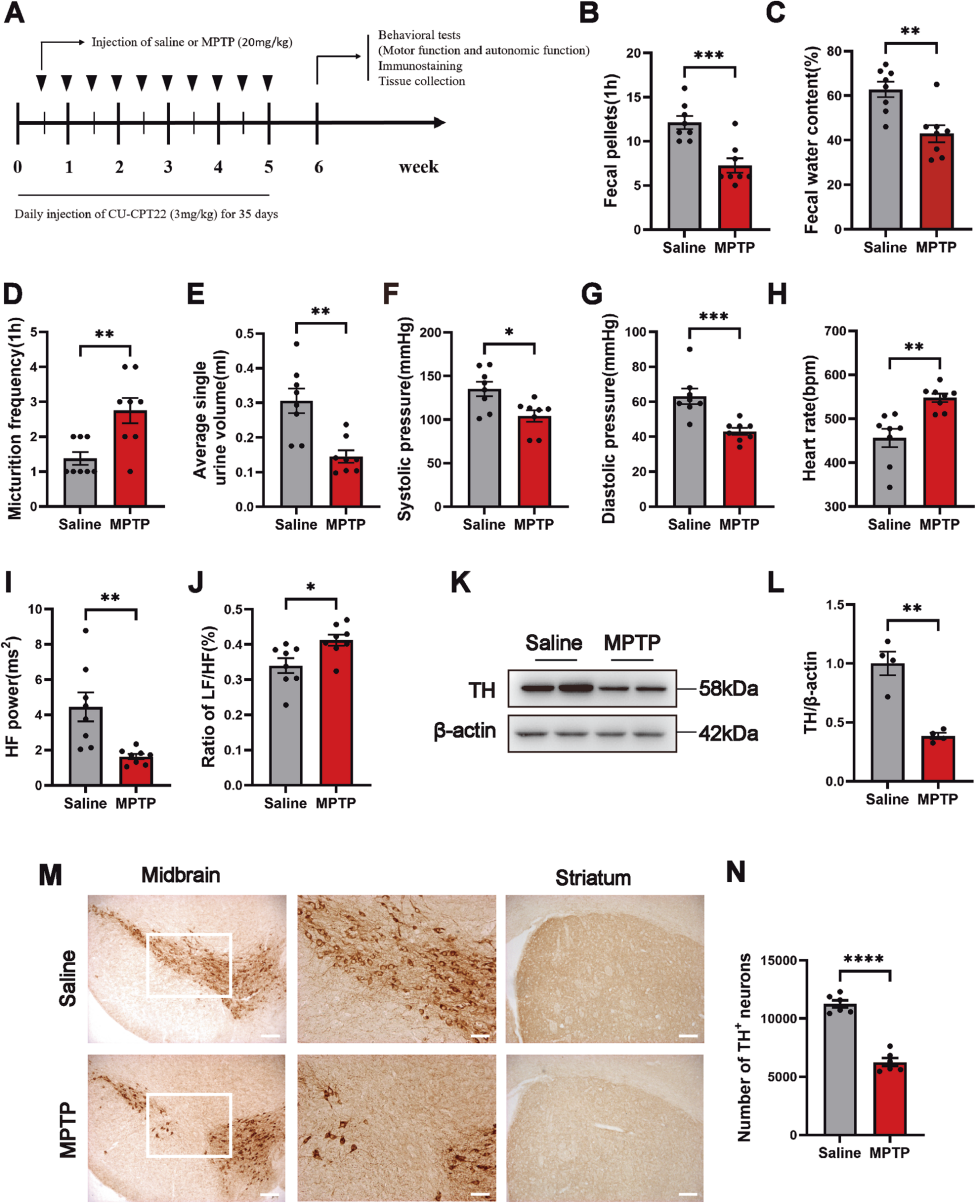
MPTP induces AutD and the loss of TH^+^ neurons in the CNS. **A** Experimental design of chronic MPTP mice model. MPTP (20 mg/kg) was injected twice a week for 5 weeks, the control group was injected with the same volume of saline, and CU-CPT22 (3 mg/kg) was injected daily for 35 days. **B**–**J** The quantitative analysis of 1 h fecal pellets, fecal water content, frequency of urination, average single urine volume, systolic pressure, diastolic pressure, heart rate, HF power, and the ratio of LF/HF in mice one week following the last MPTP injection. *n* = 8 per group. **K**, **L** Representative western blot bands and the quantitative analysis of TH level in the midbrain. *n* = 4 per group. **M**, **N** Representative immunohistochemical staining images of TH in the midbrain and striatum, and the quantitative analysis of the number of TH^+^ neurons in the midbrain, scale bar=200 μm for the left column in the midbrain, 100 μm for the right column in the midbrain and 200 μm in the striatum. *n* = 6 per group. Data were presented as mean ± SEM and analyzed using Student’s *t*-test. *P < 0.05, **P < 0.01, ***P < 0.001, ****P < 0.0001. MPTP 1-methyl-4-phenyl-1,2,3,6-tetrahydropyridine, AutD autonomic dysfunction, TH tyrosine hydroxylase, CNS central nervous system.

To explore the autonomic function phenotype in PD mice, we administered 10 injections of MPTP to construct a chronic model, and the mice were assessed one week after the last injection (Fig. 1A). Motor dysfunction in MPTP-treated mice was manifested by a reduction in the velocity and total distance in the open field, shortened latency to fall in the rotarod test, and the extension of the total time in the pole test (Fig. S1A–C). In addition, MPTP-treated mice exhibited a reduction in the number of 1 h fecal pellets with a decrease in fecal water content compared to the saline group (Fig. 1B, C), and an increase in the frequency of micturition in 1 h and a decrease in the volume of single urine output (Fig. 1D, E), which suggested an gastrointestinal dysfunction and underactive bladder in MPTP mice. As shown in Fig. 1F and G, mice in the MPTP group exhibited a decrease in systolic and diastolic blood pressure. Heart rate in the MPTP-treated mice was significantly increased (Fig. 1H). Also, analysis of the frequency domain of heart rate variability (HRV) revealed a significant decrease in HF power and an increase in the ratio of LF/HF in the MPTP-treated mice indicating a deficit in the sympathetic and parasympathetic nerve (Fig. 1I, J). As illustrated in Fig. 1K and L, compared with the saline group, the expression of TH in the midbrain declined in the MPTP group. Finally, immunohistochemistry results showed a significant reduction in the number of TH^+^ neurons in the midbrain and striatum which revealed the loss of DA neurons in the MPTP group (Fig. 1M, N).

### P-α-syn deposited in SCs upregulated the level of TLR2 accompanied by vagus nerve demyelination in the MPTP-induced mice

**Fig. 2:**
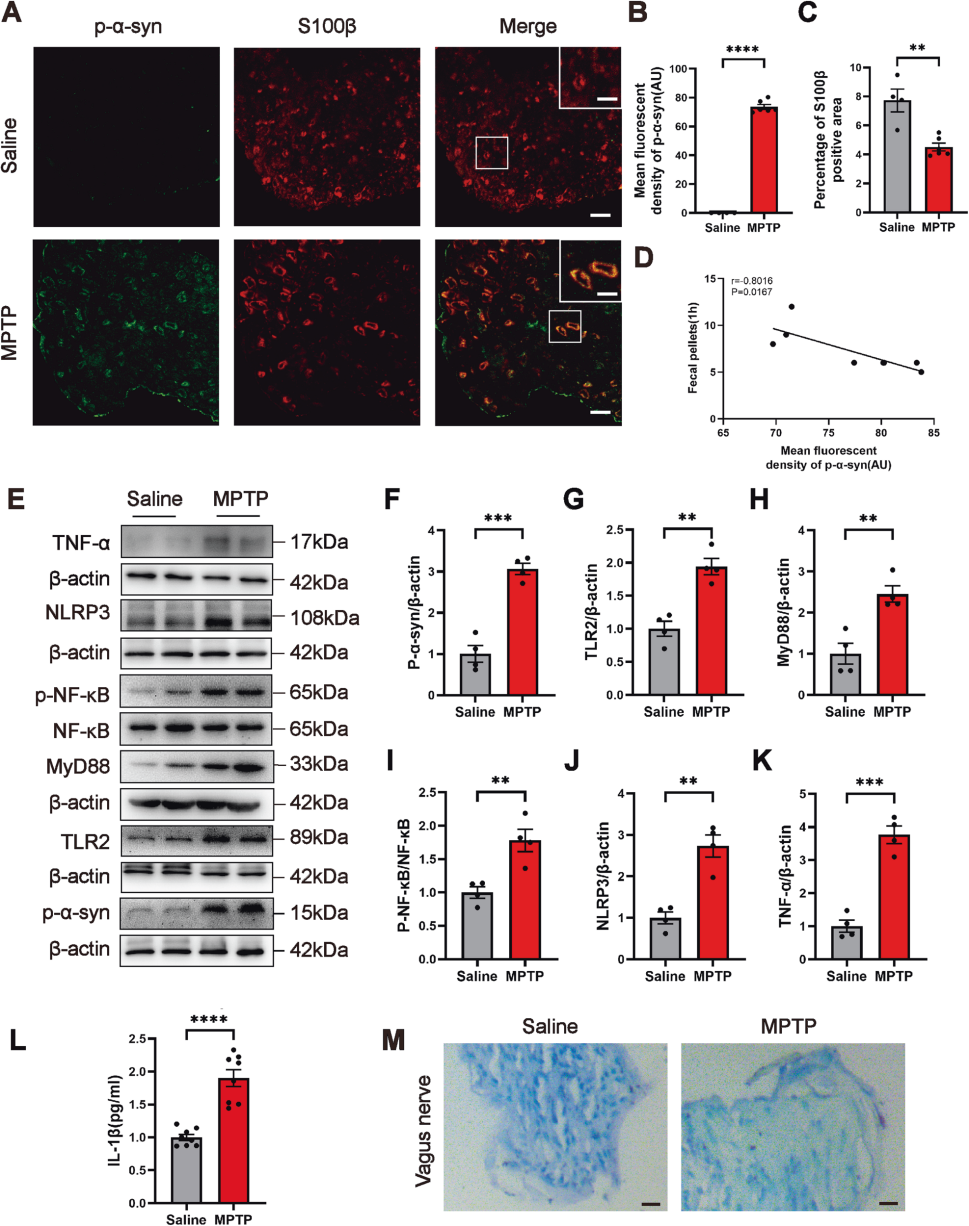
MPTP induces the deposition of p-α-syn in SCs accompanied by elevated TLR2 level and vagus nerve demyelination. **A** Representative confocal images of p-α-syn (green) and S100β (red) in the vagus nerve of mice in the saline or MPTP group, scale bar=10 μm, and enlarged scale bar=5 μm. **B**, **C** The percentage of S100β positive area and the mean fluorescent density of p-α-syn in the saline or MPTP group. *n* = 4 in the saline group and *n* = 6 in the MPTP group. **D** Pearson’s correlation analysis between the mean fluorescent density of p-α-syn in vagus nerve SCs and the 1 h fecal pellets of the MPTP mice. *n* = 8. **E** Representative western blot bands of p-α-syn, TLR2, MyD88, p-NF-κB/NF-κB, NLRP3, and TNF-α in the vagus nerve of mice in the saline or MPTP group. **F**–**K** The quantitative analysis of p-α-syn, TLR2, MyD88, p-NF-κB/NF-κB, NLRP3, and TNF-α levels in the vagus nerve. *n* = 4 per group. **L** ELISA analysis of IL-1β in the vagus nerve of saline and MPTP group. *n* = 8 per group. **M** Representative images of Luxol fast blue staining of myelin in the vagus nerve, scale bar=20 μm. Data were presented as mean ± SEM and analyzed using Student’s *t*-test. **P < 0.01, ***P < 0.001, ****P < 0.0001. p-α-syn phosphorylated α-synuclein, SCs Schwann cells, TLR2 Toll-like receptor 2.

To further interrogate whether p-α-syn deposits in the vagus nerve SCs activate TLR2 and are accompanied by vagus nerve demyelination, we employed immunofluorescence to co-label the marker of SCs S100β and p-α-syn in the vagus nerve of saline and MPTP mice to detect the deposition of p-α-syn in SCs. The findings revealed p-α-syn co-localization with S100β in the vagus nerve of MPTP mice (Fig. 2A, B), as well as a decrease in S100β positive area compared with the saline group (Fig. 2C), indicating the existence of p-α-syn in SCs and demyelination in the vagus nerve of MPTP mice. Notably, Pearson’s correlation analysis revealed a negative connection between the expression of p-α-syn in the vagus nerve of MPTP mice and the 1 h fecal pellets (Fig. 2D), implying that the deposition of p-α-syn in vagus nerve SCs is involved in the pathogenesis of AutD in PD. Furthermore, the results of western blot showed that there was a deposition of p-α-syn accompanied by an upregulation of TLR2 level in the vagus nerve of MPTP mice (Fig. 2E–G), with a similar elevation of the key junction protein molecule MyD88 in the TLR2 signaling pathway (Fig. 2E, H). Besides, the p-NF-κB expression level was also increased (Fig. 2E, I). The production of pro-inflammatory molecules such as NLRP3, TNF-α, and IL-1β increased at the same time (Fig. 2E, J–L). Finally, Luxol fast blue staining was implemented to observe the morphological changes of the vagus nerve, and as shown in Fig. 2M, compared to the saline group, the vagus nerve in MPTP mice was more sparse and exhibited nerve fibers decreased.

### P-α-syn deposited in RSC96 cells after α-synuclein preformed fibril (α-syn PFF) stimulation and involved in the loss of cell viability and cell death

**Fig. 3:**
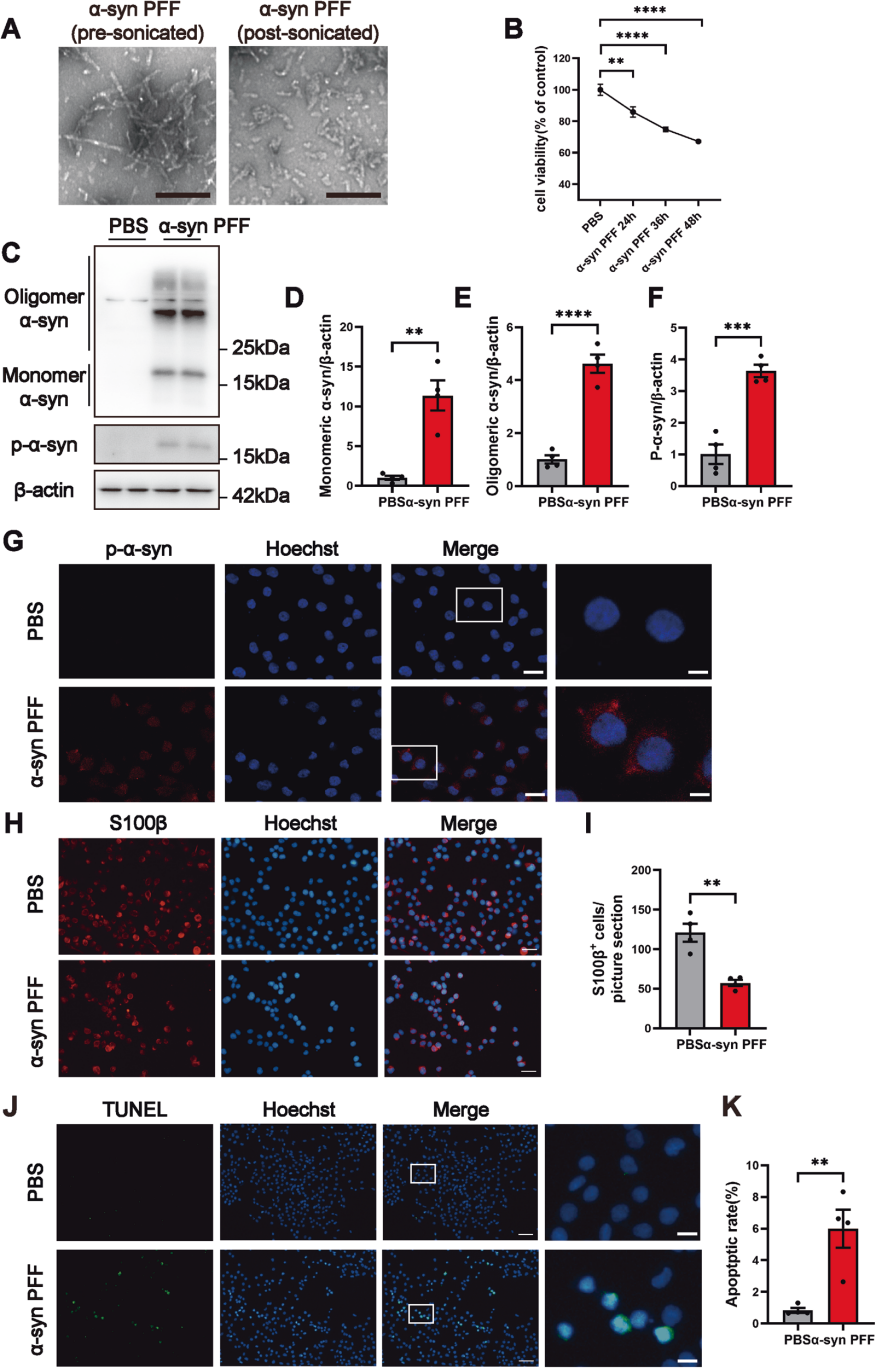
Deposition of p-α-syn after stimulation with α-syn PFF induced decreased viability and apoptosis in RSC96 cells. **A** Representative TEM images of pre-sonicated α-syn PFF and postsonicated PFF, scale bar=200 nm. **B** Cell viability at 24 h, 36 h, and 48 h after α-syn PFF stimulation was detected by CCK8 assay. *n* = 4. **C** Representative western blot bands of oligomer α-syn, monomer α-syn and p-α-syn. **D**–**F** The quantitative analysis of monomer α-syn, oligomer α-syn, and p-α-syn. *n* = 4. **G** Representative immunofluorescence images of p-α-syn in RSC96 cells stimulated with α-syn PFF for 24 h, scale bar=10 μm, enlarged scale bar=5 μm. **H**, **I** Representative images of S100β^+^ RSC96 cells stimulated with α-syn PFF for 24 h, and the quantitative analysis of cell count, scale bar=20 μm. *n* = 4. **J**, **K** Representative images of TUNEL staining, and the quantitative analysis of apoptosis rate in PBS or α-syn PFF group, scale bar=50 μm, enlarged scale bar=10 μm. *n* = 4. Data were presented as mean ± SEM and analyzed using Student’s *t*-test. **P < 0.01, ***P < 0.001, ****P < 0.0001. Cell experiments were repeated three times independently. α-syn PFF α-synuclein preformed fibril, TEM transmission electron microscopy.

For further validation of p-α-syn mediated SCs damage, the RSC96 cell line was stimulated with α-syn PFF. Pre-sonicated and post-sonicated α-syn PFF were shown by the transmission electron microscopy (TEM) (Fig. 3A). Cell viability was measured by CCK8 after 24 h, 36 h, and 48 h stimulation of α-syn PFF. The findings showed that there was a time-dependent loss of cell viability following treatment with α-syn PFF (1 µg/ml), and the stimulation time of 24 h was chosen for subsequent tests because α-syn PFF does not contribute to cell viability less than 80% (Fig. 3B). According to western blot, the levels of intracellular monomeric and oligomeric α-synuclein were increased as well as the production of p-α-syn (Fig. 3C–F). Also, the immunofluorescence assay detected the expression of p-α-syn in the α-syn PFF-treated group (Fig. 3G). Moreover, to quantify the total number of RSC96 cells after α-syn PFF treatment, cultivated cells were stained with S100β. As shown in Fig. 3H and I, the amount of RSC96 cells was significantly reduced after the treatment of α-syn PFF. Finally, apoptotic cells were detected in the α-syn PFF-treated group by TUNEL assay, and their nuclei were stained yellow-green, whereas there were almost no positive cells in the PBS-treated group (Fig. 3J, K).

### P-α-syn induced cellular inflammation by interacting with TLR2 in RSC96 cells

**Fig. 4:**
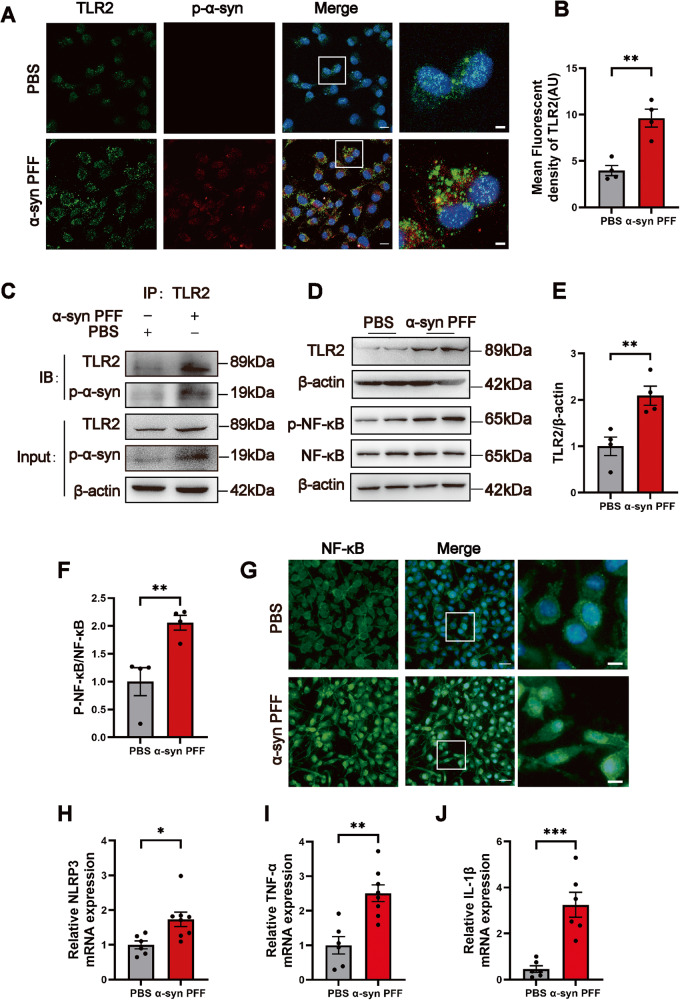
P-α-syn interacts with and activates TLR2, promoting cellular inflammatory responses in RSC96 cells. **A**, **B** Representative confocal images of TLR2 (green) and p-α-syn (red) co-localization in RSC96 cells stimulated with α-syn PFF for 24 h, and the mean fluorescent density of TLR2 in PBS or α-syn PFF group, scale bar=10 μm, enlarged scale bar=5 μm. **C** The interaction between p-α-syn and TLR2 was detected in RSC96 cells by Co-IP assay. **D**–**F** Representative western blot bands of TLR2 and p-NF-κB /NF-κB, and the quantitative analysis. *n* = 4. **G** Representative images of NF-κB nuclear translocation after stimulation of α-syn PFF for 24 h, scale bar=10 μm, enlarged scale bar=5 μm. **H**–**J** The mRNA levels of NLRP3, TNF-α, and IL-1β were quantified in RSC96 cells. *n* = 6. Data were presented as mean ± SEM and analyzed using Student’s *t*-test. *P < 0.05, **P < 0.01, ***P < 0.001. Cell experiments were repeated three times independently. Co-IP co-immunoprecipitation.

As a pattern recognition receptor, TLR2 is widely expressed in neurons and glial cells, and the activation of TLR2 can trigger a pro-inflammatory response [18]. To investigate the role of p-α-syn in mediating cell injury via TLR2 in RSC96 cells, cells were treated with PBS or α-syn PFF for 24 h. Results of immunofluorescence staining showed that p-α-syn co-localized with TLR2 in the cells of the α-syn PFF-treated group but little in the PBS-treated group (Fig. 4A). Furthermore, TLR2 fluorescence intensity was increased in the α-syn PFF group as compared to the PBS group (Fig. 4B). Interestingly, the result of Co-immunoprecipitation (Co-IP) assay revealed that there exists a significant interaction between p-α-syn and TLR2 in α-syn PFF-treated RSC96 cells (Fig. 4C). In addition, the results of western blot indicated an elevated expression of TLR2 and p-NF-κB/NF-κB in the RSC96 cells of the α-syn PFF-treated group (Fig. 4D–F), and nuclear translocation of NF-κB was detected by immunofluorescence (Fig. 4G). Furthermore, the mRNA levels of NLRP3, TNF-α, and IL-1β were elevated in the α-syn PFF group (Fig. 4H–J).

**Fig. 5:**
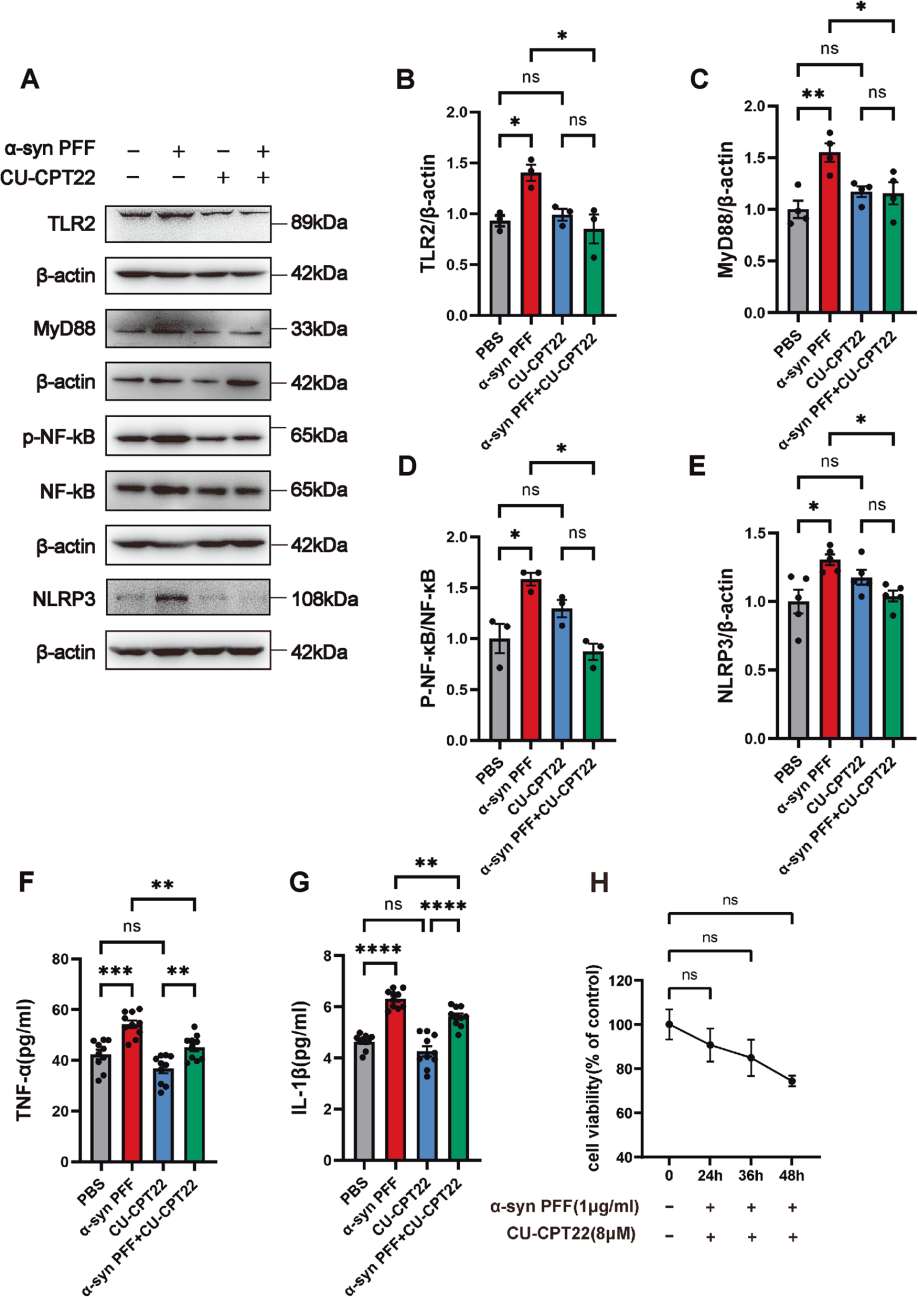
Inhibition of TLR2 reduces the p-α-syn-mediated inflammatory response in RSC96 cells. **A** Representative western blot bands of TLR2, MyD88, p-NF-κB/NF-κB, and NLRP3 of the RSC96 cells in the PBS, α-syn PFF, CU-CPT22, and α-syn PFF + CU-CPT22 groups. *n* = 4. **B**–**E** The quantitative analysis of TLR2, MyD88, p-NF-κB/NF-κB, and NLRP3. *n* = 4. **F**, **G** The levels of TNF-α and IL-1β were detected by ELISA. *n* = 10. **H** Cell viability at 24 h, 36 h, and 48 h after α-syn PFF and CU-CPT22 stimulation was detected by CCK8 assay. *n* = 6. Data were presented as mean ± SEM and analyzed by two-way ANOVA followed by Bonferroni’s multiple comparison test. *P < 0.05, **P < 0.01, ***P < 0.001, ****P < 0.0001, ns, not significant. Cell experiments were repeated three times independently.

To further explore the role of TLR2 in the process of p-α-syn-induced RSC96 cells damage, pre-treated RSC96 cells with CU-CPT22, a specific blocker of TLR2, 1 h before α-syn PFF stimulation. Western blot results showed that compared with the α-syn PFF group, TLR2 was inhibited in the α-syn PFF + CU-CPT22 group (Fig. 5A, B), while MyD88 and p-NF-κB levels (Fig. 5A, C, D) were decreased accompanied by the reduced level of NLRP3 (Fig. 5A, E). Moreover, ELISA was used to evaluate the release of inflammatory factors and showed decreased levels of TNF-α and IL-1β in the α-syn PFF + CU-CPT22 group (Fig. 5F, G). Furthermore, after the administration of CU-CPT22, the effect of α-syn PFF stimulation on the viability of RSC96 cells was not significant (Fig. 5H), indicating that CU-CPT22 greatly reduced the loss of cell viability following α-syn PFF stimulation.

### TLR2 inhibition improves AutD and protects the vagus nerve from p-α-syn*-*mediated myelin destruction in PD

**Fig. 6:**
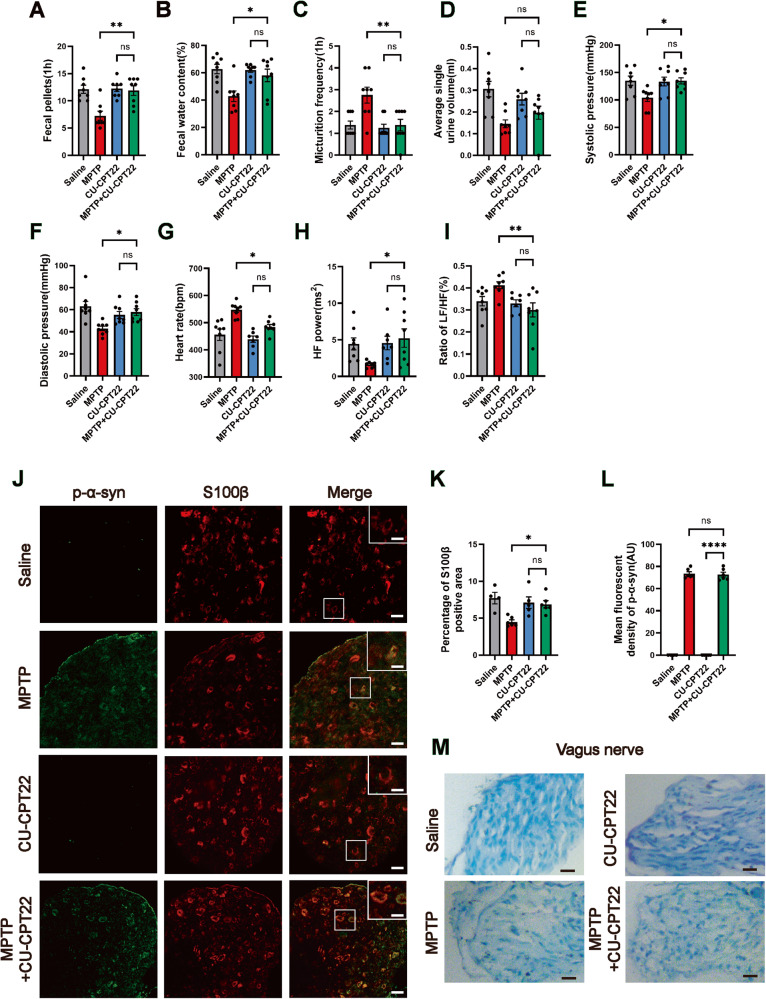
Inhibition of TLR2 improves PD AutD and alleviates p-α-syn-mediated myelin destruction of the vagus nerve. **A**–**I** The quantitative analysis of 1 h fecal pellets, fecal water content, frequency of urination, average single urine volume, systolic and diastolic blood pressure, heart rate, HF power, and the ratio of LF/HF in mice of saline, MPTP, CU-CPT22, MPTP + CU-CPT22 groups. *n* = 8. **J** Representative confocal images of p-α-syn (green) and S100β (red) in the vagus nerve of mice treated with saline, MPTP, CU-CPT22, and MPTP + CU-CPT22. **K**, **L** The percentage of S100β positive area and the mean fluorescent density of p-α-syn in four groups, scale bar=10 μm, enlarged scale bar=5 μm. *n* = 4 in the saline group, *n* = 6 in the MPTP group, *n* = 5 in the CU-CPT22 group, and n = 6 in the MPTP + CU-CPT22 group. **M** Representative images of Luxol fast blue staining of myelin in the vagus nerve, scale bar=20 μm. Data were presented as mean ± SEM and analyzed by two-way ANOVA followed by Bonferroni’s multiple comparison test. *P < 0.05, **P < 0.01, ****P < 0.0001, ns, not significant.

To further validate the critical role of TLR2 in p-α-syn-mediated SCs damage and PD AutD. Mice were injected with CU-CPT22 daily for 35 days as shown in Fig. 1A. The results showed that mice in the MPTP + CU-CPT22 group exhibited an increased number of 1 h fecal pellets and water content compared to the mice in the MPTP group (Fig. 6A, B). Consistently, the frequency of micturition declined but there was no significant variation in the volume of average single urine (Fig. 6C, D). MPTP + CU-CPT22-treated mice displayed elevated systolic and diastolic blood pressure (Fig. 6E, F), and heart rate (Fig. 6G). Analysis of the frequency domain in HRV indexed showed that in comparison to the MPTP group, the HF power was significantly increased and the ratio of LF/HF was markedly decreased in the MPTP + CU-CPT22 group (Fig. 6H, I). In addition, the motor deficits of mice in the MPTP + CU-CPT22 group were improved (Fig. S2A-C). Additionally, immunofluorescence showed an increased percentage of S100β positive area in the vagus nerve of the MPTP + CU-CPT22 group compared to the MPTP group (Fig. 6J, K), but the mean fluorescent density of p-α-syn had no significant difference between the MPTP and MPTP + CU-CPT22 groups (Fig. 6J, L). Notably, the Luxol fast blue staining of the vagus nerve in the MPTP + CU-CPT22 mice showed less nerve loss than in MPTP mice (Fig. 6M).

## Discussion

Although the prognosis of PD patients is greatly improved by dopamine replacement therapy, the non-motor symptoms are not significantly alleviated but even worse. To date, neuronal destruction and α-synuclein aggregation of central autonomic control centers including the spinal cord, brain stem, and cortex could lead to the development of AutD. However, AutD can be also attributed to the functional and structural disorders of the peripheral sympathetic nervous system and parasympathetic nervous system including the enteric neural plexuses, and they exhibit α-synuclein pathology and neuronal destruction, even ten years precede CNS degeneration, which might reflect the early neuropathology [13, 19]. Both in the early and late stages of PD, a lot of patients present with severe peripheral significant neurodegeneration [20, 21]. Evidence from clinical and animal studies confirms that the axonal degeneration of the vagus nerve is common in PD, and associated with the development of the disease [22, 23]. Here, by injecting with MPTP, we described a PD mouse model, which exhibited AutD as performed by behavioral and pathologic examinations. Our findings put forward for the first time that p-α-syn deposited in the vagus nerve SCs in chronic MPTP-treated mice and involved in the phenotype of autonomic function impairments. Furthermore, in vitro, our data revealed p-α-syn deposited in RSC96 cells directly involved in inflammatory cascade response, the loss of cell viability, and cell death by interacting with and activating TLR2. In contrast, inhibiting TLR2 with CU-CPT22 not only alleviated the inflammatory response and the loss of cell viability in vitro but also improved the AutD and vagus nerve SCs degeneration in vivo.

Autonomic nerves that innervate peripheral organs are affected by α-synucleinopathy at any stage of PD and cause peripheral nerve dysfunction. The aggregation of α-synuclein in cardiac sympathetic nerves induces sympathetic failure resulting in cardiovascular manifestations [24]. Inoculating α-syn PFF in aged mice duodenum could initiate inflammatory responses and promote gastrointestinal dysfunction [25]. Transgenic A30P-α-synuclein-overexpressing PD mice exhibit significant α-synuclein pathology in enteric neurons in the small intestine and colon before the onset of motor symptoms accompanied by a retarded bowel movement and slowed contraction [26]. In this study, MPTP mice exhibited significant autonomic deficits, manifested by constipation, urinary dysfunction, and cardiovascular disruption. Consistently, Feng Lai et al. found that chronic low doses of MPTP injection mice display gastrointestinal dysfunction and intestinal pathology, accompanied by changes in the gut microbiota [27]. The study from Xiaoli Liu showed that MPTP induced the impairment of cardiovascular function [28]. Of note, these studies did not focus on pathological changes in the vagus nerve that innervates the organs. In our study, observations from luxol fast blue and Immunofluorescence confirmed the abnormal ultrastructure of the vagus nerve performed as the loss of SCs. The findings imply that the abnormal ultrastructure of the vagus nerve gets involved in the development of AutD in PD.

SCs are peripheral glial cells that make up the myelin sheath of the vagus nerve with great plasticity. The vagus nerve also contains unmyelinated nerve fibres, such as visceral afferent fibres. In addition to maintaining axonal integrity and secreting neurotrophic factors, SCs could activate negative regulatory factors and lead nerves to act demyelinated when damaged [29]. Our previous findings showed that p-α-syn predominantly expressed in SCs and less in axons in the sural nerve of PD patients. In addition, p-α-syn was similarly detected in MPTP mice sciatic nerve SCs, and this is consistent with our observations in the vagus nerve of MPTP mice with concomitant AutD [9, 10]. A published study from Keiko Nakamura detected the accumulation of p-α-syn in SCs from patients with multisystem atrophy, revealing evidence for the involvement of SCs in the pathological process of multisystem atrophy [30]. Here, using immunofluorescence we detected a co-labeling of p-α-syn with the SCs marker S100β in MPTP-treated mice, while it was not demonstrated in the saline group, and this might be responsible for the alterations in the ultrastructure of the vagus nerve.

Extensive publications reported the status of TLRs in neurodegenerative diseases. Among these, the contribution of microglia TLRs signaling to central pathology has been widely studied [31, 32]. Similar to microglia in CNS, SCs act as sentinels in PNS and express relatively high levels of TLRs, which might be necessary for immune surveillance [33]. It has been reported that Mycobacterium leprae stimulated TLR2 and induced apoptosis in SCs, in which NF-κB acts as a transcriptional repressor with pro-apoptotic activity [34]. In addition, other studies have shown that the knockdown of TLR2 delays demyelination after nerve crush, suggesting the key role of TLR2 in nerve regeneration [35]. Our previous RNA-sequencing analysis of the mice sciatic nerve showed elevated levels of TLR2 in MPTP mice [10]. Consistently, in our study, TLR2 is activated and mediated massive pro-inflammatory responses in MPTP mice vagus nerve SCs, in addition to p-α-syn deposition. Interestingly, we found an interaction between p-α-syn and TLR2 at the cellular level, which activated the TLR2 signaling pathway and induced nuclear translocation of NF-κB, finally mediating apoptosis and the release of inflammation. A study published in 2021 pointed out that selectively targeting the TLR2/MyD88/NF-κB signaling pathway could inhibit the propagation of α-synuclein in vitro and in vivo, revealing that the spreading of α-synuclein dependents on the TLR2/MyD88/NF-κB signaling pathway [36]. Furthermore, Changyoun Kim et al. found the oligomeric α-synuclein released by neurons acts as an endogenous agonist of microglia and activates microglia paracrine secretion [17].

We employed CU-CPT22 to suppress TLR2 in vivo and in vitro, respectively, and demonstrated that TLR2 activation in vagal SCs underlies the onset of AutD. As a novel small molecule compound, CU-CPT22 can compete with the synthetic triacylated lipoprotein (Pam3CSK4) binding to TLR1/2 with high inhibitory activity and specificity [37]. In this study, results showed that there was no significant loss of RSC96 cell viability in the α-syn PFF + CU-CPT22 group compared to the α-syn PFF group after 24 h, 36 h, and 48 h of stimulation. In addition, CU-CPT22 treatment reduced the levels of NLRP3, TNF-α, and IL-1β. The majority of the autonomic functions were consistently improved in the mice of the MPTP + CU-CPT22 group. According to our findings, MPTP + CU-CPT22 group mice displayed recovered gastrointestinal symptoms and a decline of micturition frequency in 1 h compared to the mice in the MPTP group, however, there was no significant difference in the volume of average single urine between the mice in the MPTP and MPTP + CU-CPT22 groups. It is worth mentioning that whether the vagus nerve innervates the kidneys is debatable [38], therefore alternative pathways may influence the reabsorption process of the renal tubules and thus urine production. Our results also show CU-CPT22 does not lower the level of p-α-syn in vagus nerve SCs, indicating p-α-syn already deposited before TLR2 activation and can’t promote p-α-syn degradation, further supporting our conclusion that p-α-syn interacts with TLR2 to induce SCs damage. It is worth noting that the current study concentrated on the role of SCs in PD AutD and the underlying mechanisms using vivo and vitro models. The findings indicate that p-α-syn interacts with TLR2 to mediate SCs damage, whereas it is needed for further investigation about the effect of TLR2 inhibition on p-α-syn transmission between SCs or SCs and neurons. In addition, more PD models should be used to expand the study of the mechanisms of autonomic dysfunction.

In conclusion, our findings reveal for the first time that p-α-syn interacts with TLR2 to induce vagus nerve SCs damage, promoting PD AutD. We shed light on insight on the probable mechanism of PD AutD and propose that inhibition of TLR2 serves as a way to reduce p-α-syn-mediated SCs damage in the vagus nerve and prevent the development of PD AutD.

## Materials and Methods

### Animals

C57BL/6 mice (male, 4-month-old, 27–32 g) were purchased from Nanjing Medical University Laboratory Animal Center (Nanjing, Jiangsu, China) and kept in the Animal Core Facility of Nanjing Medical University under a standard 12 h light cycle. Water and food were supplied ad libitum. Healthy male mice of equal weight and 4 months of age were chosen for the experiment. Experimental protocols were implemented strictly following the guidelines of the Institutional Animal Care and all animal-related experiments were approved by the Institutional Animal Care and Use Committee of the Nanjing Medical University Experimental Animal Department. The mice were divided into control (*n* = 8), MPTP (*n* = 8), CU-CPT22 (*n* = 8), and MPTP + CU-CPT22 (*n* = 8) groups using a random number table. MPTP (Cat#S4732, Selleck, Houston, Texas, USA, 20 mg/kg) was injected twice a week for 5 weeks, and an equal amount of saline was administered to the control group. CU-CPT22 (Cat#HY-108471, MCE, New Jersey, USA, 3 mg/kg) was administered daily for 35 days. Behavioral testing was performed and tissue was collected 7 days after the last injection.

### Cell culture and treatment

The RSC96 rat SCs line was purchased from Procell Life Science & Technology (Wuhan, China), and they are primary cultured rat SCs that spontaneously transformed after extensive cultivation. Besides, mycoplasma identification by Hoechst staining, PCR, and routine culture techniques was negative, and the RSC96 cell line has been certified by ATCC and analyzed by STR. Cells were seeded in Petri dishes maintained in Dulbecco’s modified Eagle’s medium (Cat#PM150210, Procell) containing 10% fetal bovine serum (Excell Bio, Shanghai, China) and 1% penicillin/streptomycin in 5% CO_2_ humidified atmosphere at 37 °C. The medium was replaced every 2 days. RSC96 cells were treated with α-syn PFF for 24 h, 36 h, and 48 h. To assess the role of TLR2, treated cells with the small molecule TLR1/2 inhibitor CU-CPT22 (8 µM) 1 h before α-syn PFF stimulation [38–40].

### Behavioral assessments

To measure behavioral deficits in MPTP-injected mice, all animals were tested for autonomic function and locomotor abilities one week after the injections, and the experimenters were double-blind in their group classification.

### Fecal collection and water content

1 h stool collection test was evaluated one week after the last injection as described before [41]. Mice fasted for 24 h and then given sufficient food within 2 h before the test. The mouse was placed individually in a clear plastic cage without water or food and was observed for defecation during the 1 h collection period. Each collection tube was weighed before and after fecal loading. Then, the fecal was immediately collected, counted, and weighed. Weighed dry weight after being dried overnight at 65 °C. The fecal water content was analyzed as follows: fecal water content (%) = [(wet weight - dry weight) / wet weight] × 100% [42].

### Urinary function measurement

#### Urine collection

The frequency of 1 h urine output and the average volume of single urination were quantified to assess the urinary function of PD mice. Mice were positioned beneath a glass with space for regular breathing and a piece of filter paper underneath. Timing began as soon as the mice were put inside the glass. Once the mice had urinated, the filter paper was removed and replaced with a fresh one right away. Then, cut along the edge of the filter paper that has been saturated with urine.

### Urine volume calculation

The urine was simulated with saline. The dry weight of the filter paper was measured against the volume of saline, and the linear correlation equation was as follows: single urine volume (ml)=0.89 × Dry weight of filter paper (g) + 0.0189.

### Cardiovascular function assessment

#### Systolic and diastolic pressure

Systolic and diastolic pressure were measured by a noninvasive tail-cuff BP analyzer (Visitech BP-2000, TX, USA) following the manufacturer’s instructions. Morning blood pressure measures were taken in all groups of mice. Each mouse’s blood pressure was monitored three times, and the mean was calculated [43].

### HRV and heart rate

HRV provides a reliable approach to evaluating the function of sympathetic and parasympathetic nerves both in the temporal and frequency domains. HRV was evaluated as previously mentioned [44]. Mice were placed on a 37 °C insulated pad after being anesthetized using a gas anesthesia machine. A biopotential electrode was used to analyze the ECG, and a powerlab26T data acquisition system was used to gather the images. Using LabChart software, the generally steady ECG images within 5 min were examined. The frequency domains such as HF and LF/HF, as well as their HR, were all recorded simultaneously.

### Motor function assessment

#### Open field test

The open field test was employed to gauge the mice’s capacity for locomotion. First, mice were placed in the center of the black box. After that, their trajectory was examined over 5 min. Finally, the total distance as well as the average speed were assessed using Openfield software (CleverSys Inc., VA, USA).

### Rotarod test

To assess the motor phenotypes of balance and coordination deficits in mice, the rotarod test was performed [45]. Each mouse was trained on the apparatus (Jiliang Pharmaceutical Engineering Co, Shanghai, China) for 3 rounds of 300 seconds at a rotational speed of 5 to 10 rpm three days before starting the experiment. After training, the rotarod test was conducted at a speed that increased consistently over 300 seconds, from 5 to 30 rpm. Then, the time each mouse dropped and the rotational speed of the rotating rod during the fall was recorded.

### Pole test

Mouse locomotor activity including bradykinesia and balance was assessed using the pole test. The procedure was implemented as described before [27, 46]. Animals were trained on a wooden pole 3 times. Each mouse’s total time to travel from the top to the bottom was timed during the pole test.

### Immunofluorescence and immunohistochemistry

After being dewaxed and rehydrated, the paraffin-embedded mice vagus nerve section (5 µm) was antigenically repaired using citrate at 100 °C for 50 min. RSC96 cells were seeded on 24-well glass slides. Then, broke the membrane with 0.3% PBST for 10 min and closed sections with 3% BSA for 2 h. Next, incubated the sections separately with anti-alpha-synuclein (Cat#ab280377, 1:500, Abcam, United Kingdom), anti-phospho S129 alpha-synuclein (Cat#ab51253, 1:500, Abcam), anti-S100β (Cat#ab52642, 1:500, Abcam), anti-NF-κB (Cat#8242, 1:500, CST, Boston, USA), and anti-TLR2 (Cat#ab209216, 1:500, Abcam) at 4 °C overnight. The following day, washed the sections with PBS 3 times and then incubated with secondary antibodies (Alexa Fluor conjugates, Abcam) for 1.5 h at room temperature. At last, the Hoechst (Cat#4082, 1:1000, CST) was stained. Images were taken with the laser scanning confocal fluorescence microscope (CarlzeissLSM710, Oberkochen, Germany).

Mice brain sections (20 µm) were incubated with 3% H_2_O_2_ for 15 min in the dark, which inactivated endogenous peroxidase. Secondary antibodies (Cat#SA00001-1, Cat#SA00001-2, 1:1000, Proteintech, Chicago, USA) were kept at room temperature for 1.5 h. After staining with DAB, dehydrated under gradient alcohol and sealed with neutral gum. Positive cells were counted serologically by Microbrightfield Stereo-Investigator software.

### Western blot

To guarantee a double-blind experiment, all tubes were changed to numbers. Lysis of tissues or cells using RIPA lysate containing protein phosphatase inhibitors. After centrifuging at 12,000 rpm for 15 min at 4 °C, the supernatant was gathered. Quantificated the protein concentration using BCA. Then added the loading buffer and heated the sample at 95 °C for 10 min. Protein samples were electrophoresed through 8–15% SDS-PAGE gel before being transferred to PVDF membranes. After blocking protein with 5% skim milk at room temperature for 2 h, PVDF membranes were incubated with the following primary antibodies at 4 °C overnight: anti-alpha-synuclein (Cat#ab280377, 1:1000, Abcam), antiphospho S129 alpha-synuclein (Cat#ab51253, 1:1000, Abcam), anti-S100β (Cat#ab52642, 1:1,000, Abcam), anti-TLR2 (Cat#ab209216, 1:1,000, Abcam), anti-NLRP3 (Cat#AG-20B-0014, 1:1,000, AdipoGen, San Diego, USA), antiphospho NF-κB (Cat#8242, 1:1000, CST), anti-MyD88 (Cat#4283, 1:1000, CST), anti-NF-κB (Cat#8242, 1:500, CST), and anti-β-actin (Cat#66009-1-Ig, 1:5000, Proteintech). The HRP-conjugated secondary antibodies were then incubated for 1.5 h at room temperature: goat anti-Mouse (Cat#SA00001-1, 1:5000, Proteintech) and goat anti-Rabbit (Cat#SA00001-2, 1:5000, Proteintech). Finally, bands were visualized by an ECL assay kit and Tanon 5200 Automatic Chemiluminescence Imaging Analysis System.

### Enzyme-linked immunosorbent assay (ELISA)

The IL-1β and TNF-α levels of vagus nerve and RSC96 cells were measured using commercial ELISA kits (IL-1β: Cat# BY-ER330206, TNF-α: BY-ER331063, Byabscience, Nanjing, Jiangsu, China) according to the manufacturer’s program.

### Real-time PCR

Total intracellular RNA was isolated from RSC96 cells using Total RNA Extraction Reagent (Cat#R401-01, Vazyme, Nanjing, Jiangsu, China). After that, the RNA was then reverse-transcribed into cDNA. Next, qPCR was conducted using ChamQ SYBR qPCR Master Mix (Cat#Q341-02, Vazyme). Finally, analysis was performed using 2^-ΔΔct^. The primer sequences of RT-qPCR are displayed in Table S1.

### Preparation of α-syn PFF

Recombinant mouse wild-type α-synuclein was prepared as previously mentioned [47]. Endotoxin resulted in less than 0.12 EU/ml by using the ToxinEraser™ Endotoxin Removal Kit. The monomeric α-synuclein was diluted to 5 mg/ml by PBS and shaken at 37 °C, 1,000 rpm for 7 days. α-syn PFF was sonicated 6 times at 20% power (1 s on and 1 s off for a total of 10 seconds) with a probe-tip sonicator before use. For experiments in RSC96, we used a concentration of 1 µg/ml unless otherwise specified, and equal amounts of PBS were added to the control group [48]. Finally, the TEM images of pre-sonicated and post-sonicated α-syn PFF were taken by a JEM 1400 TEM (JEOL, Tokyo, Japan).

### Cell viability assays

Cell viability was evaluated using the CCK8 (Cat#K1018-30, APExBIO, Houston, USA) assay in accordance with the manufacturer’s instructions. Briefly, RSC96 cells were plated in 96-well plates at a density of 2 × 10^4 ^mL^−1^ per well until cells were in good growth condition, then stimulated α-syn PFF (1 µg/ml) for 24 h, 36 h, and 48 h. Mixed CCK8 and medium at 1:10. Finally, the OD value at 450 nm was measured using a microplate reader (FLx800™, Bio-Tek, Vermont, USA), and the data were presented as a percentage of the control [49].

### Apoptosis analysis

TUNEL assay was used to detect apoptosis commonly. Here, we used the In Situ Cell Apoptosis Detection Kit, FITC (Cat# E607178, Sangon Biotech, Shanghai, China) to detect RSC96 cell apoptosis after α-syn PFF treatment according to the manufacturer’s recommendations. Cells were fixed with 4% PFA at room temperature for 30 min. The latter procedure was performed according to the manufacturer’s instructions. Finally, DAPI (Cat#D9542, Sigma-Aldrich, St. Louis, Missouri, USA) was used for staining nuclei.

### Co-IP assay

Co-IP was performed to identify the interaction between p-α-syn and TLR2, which was conducted by the previous process [50]. Firstly, cellular protein samples were extracted and quantified, then antibodies were added and incubated protein-antibody complexes at 4 °C overnight. Protein A/G beads (Cat#88808, Thermo Fisher, Massachusetts, USA) were used to precipitate protein-antibody complexes and then dissolved in SDS-PAGE. The subsequent process was the same as the western blot analysis.

### Statistical analysis

For statistical analysis, the GraphPad Prism 9.0 software was applied. All data were represented as mean ± SEM. The data were regularly distributed, with uniform variance. All results shown in the figure are representative of at least three independent experiments. Student’s t-test was used to compare data between two groups, and one-way analysis of variance (ANOVA) or two-way ANOVA followed by Bonferroni’s multiple comparison test was used to compare data between three or more groups. Pearson’s correlations were used to investigate the relationship between p-α-syn level in vagus nerve SCs and 1 h fecal pellets. *P* < 0.05 was indicated as a statistical significance.

### Supplementary information

Supplementary Figure Legents

Supplemental Figure S1

Supplemental Figure S2

Supplementary Table S1

Original Data File

## Data Availability

The datasets used and analyzed during the current investigation are accessible upon reasonable request from the corresponding author.
