# Supplementary material for: Phosphorylated α-synuclein deposited in Schwann cells interacting with TLR2 mediates cell damage and induces Parkinson’s disease autonomic dysfunction
Source: Cell Death Discov. 2024 Jan 26;10:52. doi: 10.1038/s41420-024-01824-8 (PMC10817950; doi:10.1038/s41420-024-01824-8)
Supplement: Supplementary file 1 — Supplementary Figure Legents [file 41420_2024_1824_MOESM1_ESM.docx]

**Fig S1. Motor deficits in MPTP-induced mice.** **A** Quantitative analysis of velocity in full and total distance of mice in the saline or MPTP group in the open field test. n=8. **B-C** Quantitative analysis of latency to fall in rotarod test and the T-TLA in pole test. n=8. Data were presented as mean ± SEM and analyzed using Student’s *t*-test. ***P*<0.01, ****P*<0.001. **Abbreviations:** T-TLA, time to climb down.

**Fig S2. Motor deficits in MPTP-induced mice were improved after treatment with CU-CPT22. A** Quantitative analysis of velocity in full and total distance of mice in the saline, MPTP, CU-CPT22, or MPTP+CU-CPT22 group in the open field test. n=8. **B-C** Quantitative analysis of latency to fall in rotarod test and the T-TLA in the saline, MPTP, CU-CPT22, or MPTP+CU-CPT22 group in pole test. n=8. Data were presented as mean ± SEM and analyzed by two-way ANOVA followed by Bonferroni’s multiple comparison test. **P*<0.05, ***P*<0.01, ns, not significant.
