# Supplementary figures and images for: Phosphorylated α-synuclein deposited in Schwann cells interacting with TLR2 mediates cell damage and induces Parkinson’s disease autonomic dysfunction
Source: Cell Death Discov. 2024 Jan 26;10:52. doi: 10.1038/s41420-024-01824-8 (PMC10817950; doi:10.1038/s41420-024-01824-8)

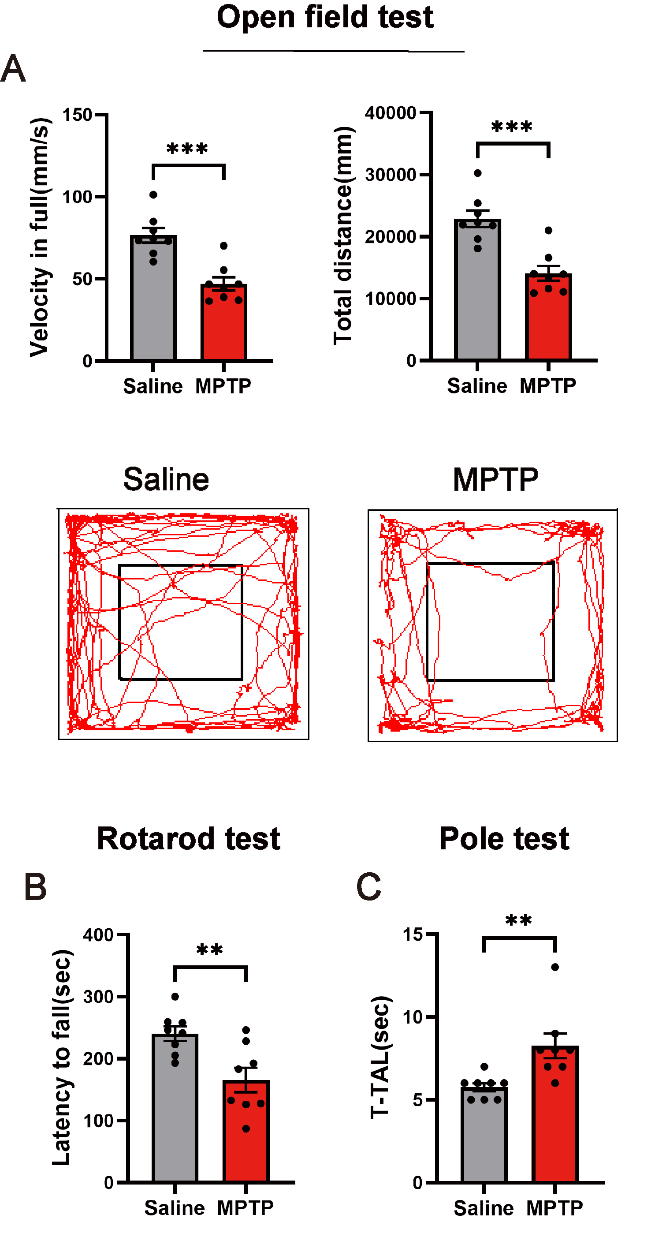

Supplement: Supplementary file 2 — Supplemental Figure S1 [file 41420_2024_1824_MOESM2_ESM.tif]

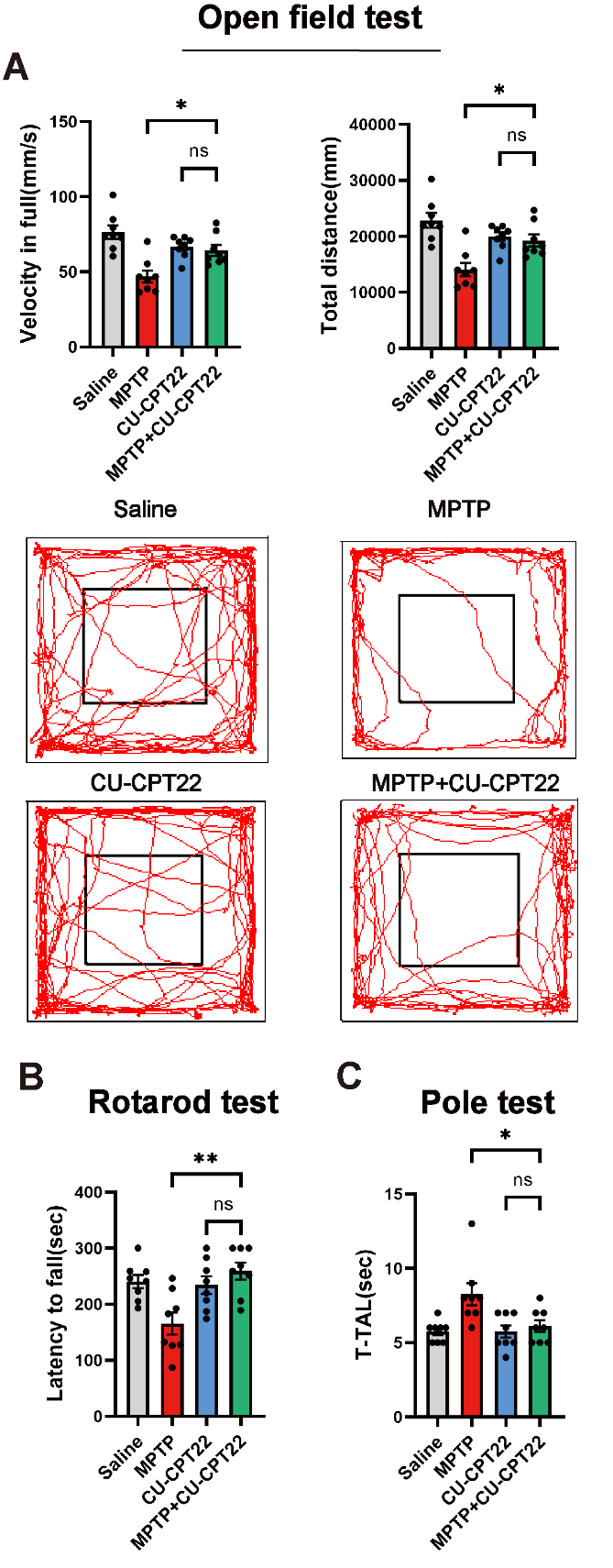

Supplement: Supplementary file 3 — Supplemental Figure S2 [file 41420_2024_1824_MOESM3_ESM.tif]
