# Supplementary material for: Phosphorylated α-synuclein deposited in Schwann cells interacting with TLR2 mediates cell damage and induces Parkinson’s disease autonomic dysfunction
Source: Cell Death Discov. 2024 Jan 26;10:52. doi: 10.1038/s41420-024-01824-8 (PMC10817950; doi:10.1038/s41420-024-01824-8)
Supplement: Supplementary file 4 — Supplementary Table S1 [file 41420_2024_1824_MOESM4_ESM.docx]

**Table S1. Primer sequences of RT-qPCR**

| Gene ID | Forward primer | Reverse primer |
| --- | --- | --- |
| NLRP3  TNF-α  IL-1β  GAPDH | AGTGGATAGGTTTGCTGGGATA  TGCCTCAGCCTCTTCTCATT  TGAAGCAGCTATGGCAACTG  TGAAGGTCGGTGTCAACGGATTTGGC | CTGGGTGTAGCGTCTGTTGAG  TGTGGGTGAGGAGCACATAG  TGCCTTCCTGAAGCTCTTGT  CATGTAGGCCATGAGGTCCACCAC |

**Note:** RT-qPCR, reverse transcription quantitative polymerase chain reaction; ID: identification; NLRP3: Nucleotide-binding oligomerization domain, leucine-rich repeat and pyrin domain- containing 3; TNF-α: tumor necrosis factor; IL-1β: Interleukin-1β; GAPDH, glyceraldehyde-phosphate dehydrogenase.
