# Supplementary material for: Phosphorylated α-synuclein deposited in Schwann cells interacting with TLR2 mediates cell damage and induces Parkinson’s disease autonomic dysfunction
Source: Cell Death Discov. 2024 Jan 26;10:52. doi: 10.1038/s41420-024-01824-8 (PMC10817950; doi:10.1038/s41420-024-01824-8)

Figure 1

TH β-actin

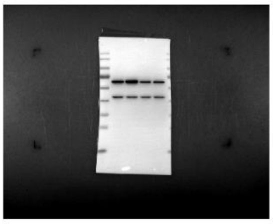

Figure 2

p-α-synuclein

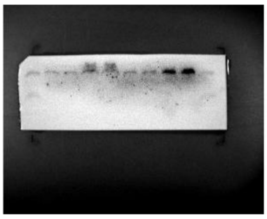

β-actin

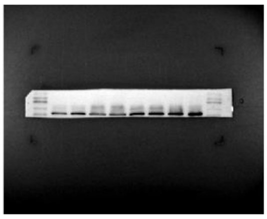

TLR2

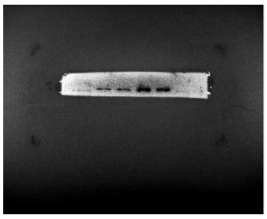

β-actin

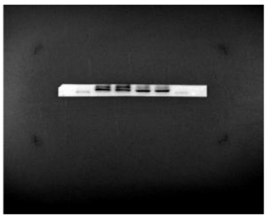

MyD88

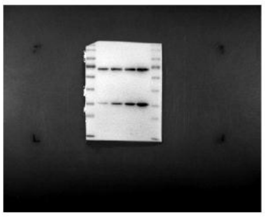

β-actin

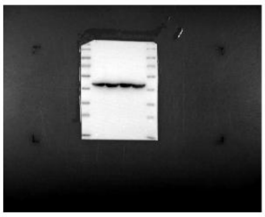

p-NF-κB

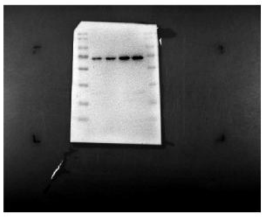

NF-κB

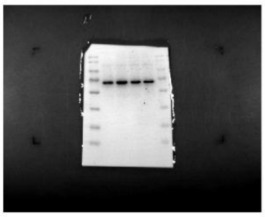

β-actin

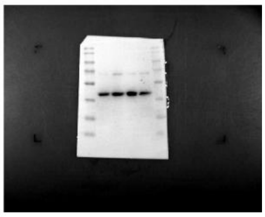

NLRP3

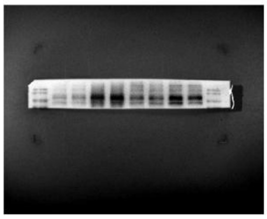

β-actin

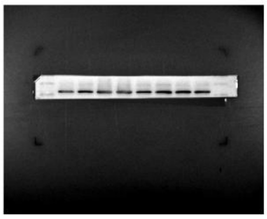

TNF-α

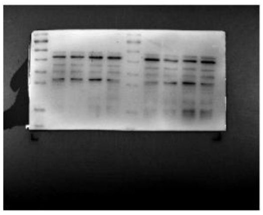

β-actin

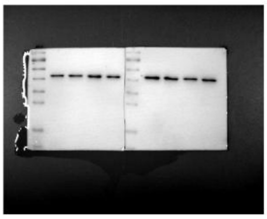

Figure 3

α-synuclein

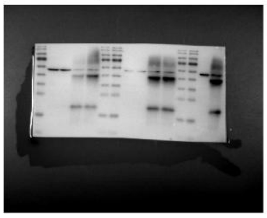

p-α-synuclein

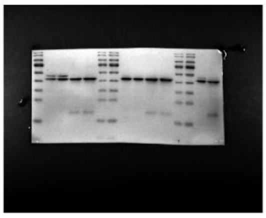

β-actin

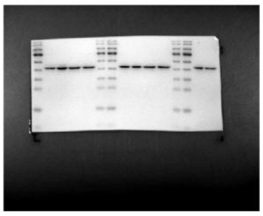

Figure 4

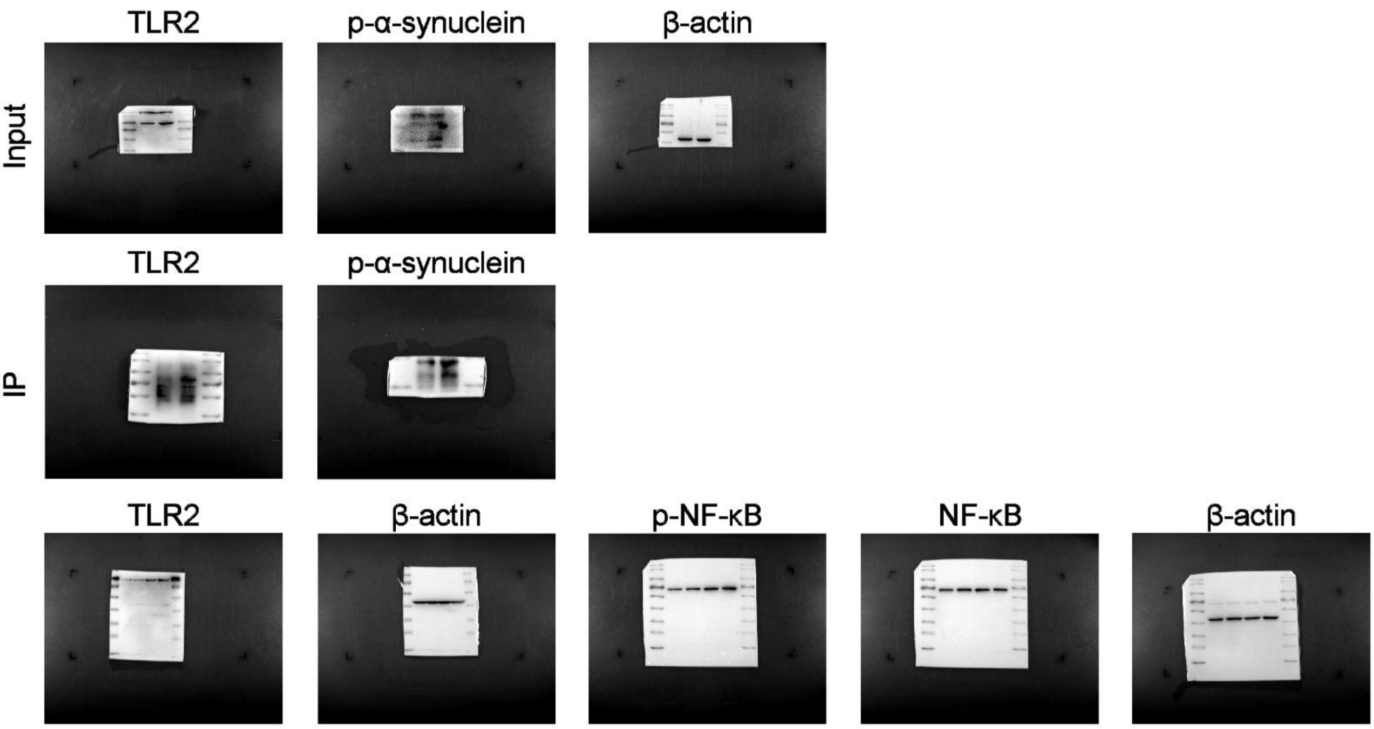

Figure 5

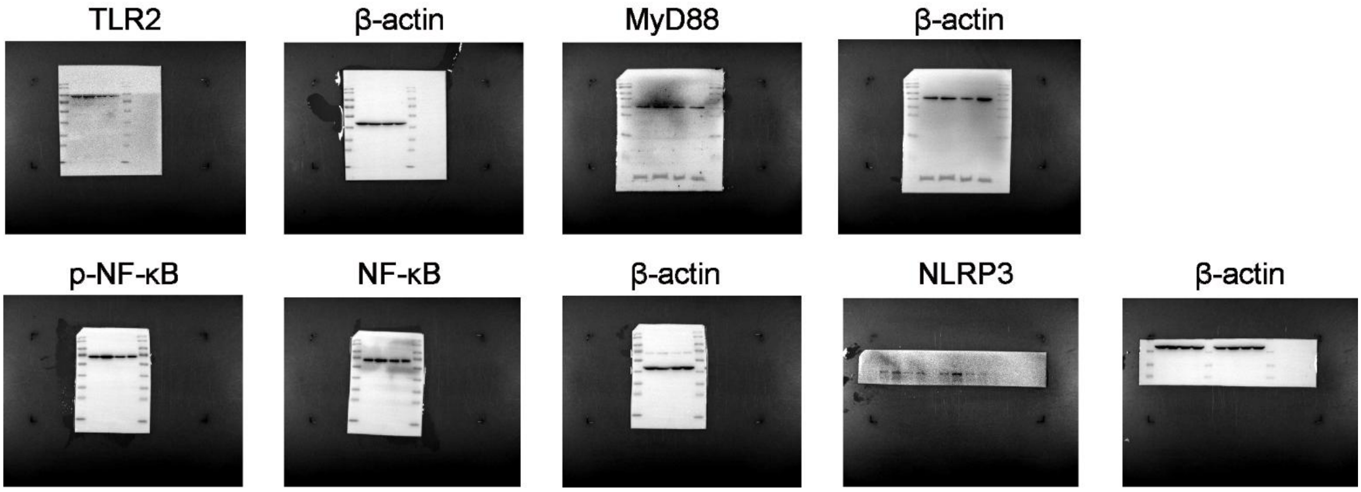

Supplement: Supplementary file 5 — Original Data File [file 41420_2024_1824_MOESM5_ESM.pdf]
